# Bionic Upper Limb Reconstruction: A Valuable Alternative in Global Brachial Plexus Avulsion Injuries—A Case Series

**DOI:** 10.3390/jcm9010023

**Published:** 2019-12-20

**Authors:** Laura A. Hruby, Clemens Gstoettner, Agnes Sturma, Stefan Salminger, Johannes A. Mayer, Oskar C. Aszmann

**Affiliations:** 1 Department of Orthopaedics and Trauma Surgery, Medical University of Vienna, Spitalgasse 23, 1090 Vienna, Austria; 2 Clinical Laboratory for Bionic Extremity Reconstruction, Medical University of Vienna, Spitalgasse 23, 1090 Vienna, Austria; clemens.gstoettner@meduniwien.ac.at (C.G.); agnes.sturma@meduniwien.ac.at (A.S.); stefan.salminger@meduniwien.ac.at (S.S.); johannes.mayer@meduniwien.ac.at (J.A.M.); oskar.aszmann@meduniwien.ac.at (O.C.A.); 3 Department of Bioengineering, Imperial College London, London SW7 2AZ, UK; 4 Division of Plastic and Reconstructive Surgery, Department of Surgery, Medical University of Vienna, Spitalgasse 23, 1090 Vienna, Austria; 5 Department of Hand, Plastic, Reconstructive and Burn Surgery, BG Unfallklinik Tuebingen, Eberhard Karls University Tuebingen, Schnarrenbergstraße 95, 72076 Tübingen, Germany

**Keywords:** brachial plexus injury, nerve root avulsion, prostheses and implants, bionics, artificial limbs, chronic pain, prosthesis fitting

## Abstract

Global brachial plexopathies including multiple nerve root avulsions may result in complete upper limb paralysis despite surgical treatment. Bionic reconstruction, which includes the elective amputation of the functionless hand and its replacement with a mechatronic device, has been described for the transradial level. Here, we present for the first time that patients with global brachial plexus avulsion injuries and lack of biological shoulder and elbow function benefit from above-elbow amputation and prosthetic rehabilitation. Between 2012 and 2017, forty-five patients with global brachial plexus injuries approached our centre, of which nineteen (42.2%) were treated with bionic reconstruction. While fourteen patients were amputated at the transradial level, the entire upper limb was replaced with a prosthetic arm in a total of five patients. Global upper extremity function before and after bionic arm substitution was assessed using two objective hand function tests, the action research arm test (ARAT), and the Southampton hand assessment procedure (SHAP). Other outcome measures included the DASH questionnaire, VAS to assess deafferentation pain and the SF-36 health survey to evaluate changes in quality of life. Using a hybrid prosthetic arm mean ARAT scores improved from 0.6 ± 1.3 to 11.0 ± 6.7 (*p* = 0.042) and mean SHAP scores increased from 4.0 ± 3.7 to 13.8 ± 9.2 (*p* = 0.058). After prosthetic arm replacement mean DASH scores improved from 52.5 ± 9.4 to 31.2 ± 9.8 (*p* = 0.003). Deafferentation pain decreased from mean VAS 8.5 ± 1.0 to 6.7 ± 2.1 (*p* = 0.055), while the physical and mental component summary scale as part of the SF-36 health survey improved from 32.9 ± 6.4 to 40.4 ± 9.4 (*p* = 0.058) and 43.6 ± 8.9 to 57.3 ± 5.5 (*p* = 0.021), respectively. Bionic reconstruction can restore simple but robust arm and hand function in longstanding brachial plexus patients with lack of treatment alternatives.

## 1. Introduction

High-speed motor vehicle accidents account for the majority of adult traumatic brachial plexus injuries (BPIs), as severe traction on the brachial plexus may occur with violent arm motion when the motorcycle rider collides with a car or other obstacle [1,2,3]. Timely primary reconstructive surgeries include direct neural repair using nerve grafts [4] as well as intra- and extraplexual nerve transfers [5], which have significantly improved functional outcomes during the past decades, especially for the shoulder and elbow [4,6,7,8,9]. 

Avulsions of multiple nerve roots, however, still have a very remote chance of recovery [10]. In some patients with global brachial plexopathy the extent of neurological injury results in complete paralysis of all upper limb muscles despite primary and secondary biological reconstructions. After long-standing denervation muscle fibrosis and joint stiffness inevitably occur. Typically, patients who regain no arm and hand function emotionally detach from their insensate, functionless limb [11]. Besides marked functional disability [12] and greatly impaired quality of life [13], a chronic pain syndrome affects up to 90% of patients with nerve root avulsions, referred to as deafferentation pain [14].

Following complex brachial plexus injuries, nerve regeneration often leads to partial re-innervation of muscles in the affected arm. Although without clinical significance to the patient, faint muscle activity may therefore still be detectable with transcutaneous electromyographic (EMG) sensors [15]. Recently, it has been shown that this residual myoactivity suffices to translate into dexterous prosthetic hand control after elective amputation and prosthetic replacement of the functionless plexus hand, a concept today known as bionic reconstruction [16]. So far, it has only been described for the transradial level in BPI patients [15,16]. Prerequisites were a sufficient shoulder and elbow function to move the prosthetic hand in three-dimensional space as well as two separable EMG signals in the forearm to reliably control the prosthetic hand [15]. Patients with global brachial plexus avulsion injuries, however, may lack useful shoulder and elbow function as well as detectable myoactivity in their forearm muscles. 

In such cases, faint muscle activity may be present more proximally in the upper arm and/or shoulder girdle. Oftentimes, however, in cases of global brachial plexus avulsion injuries, no contractile function can be elicited with needle EMG testing or even upon intra-operative nerve stimulation. This is explained by the fact that all muscle fibres have been lost and replaced with adipose and fibrous connective tissue with long-standing denervation [17]. A limited number of functional axons, however, may still be present within the respective muscle branches of the cardinal upper limb nerves, amenable for nerve transfer surgery. With an intra-operative fast staining technique developed in our group nerve biopsies can therefore be screened for single fascicles containing viable motor axons [15]. These few motor axons may then be used to re-innervate a free muscle transplant transferred to the patient′s arm, which will not have the functional capacity or power of a biological muscle but instead serve as an additional EMG signal for future prosthetic control (given that a sufficient number of axons regenerate into the transferred muscle target). Since the dexterity of prosthetic control increases with the number of available EMG signals [18,19], free functional muscle transfers have previously been used to increase the number of these muscle signals [16].

Another possibility to improve the biotechnological interface linking the patient to the prosthesis is the surgical rearrangement of muscle locations to facilitate signal uptake and reduce signal crosstalk within the future prosthetic socket. This is especially relevant if innervated muscles are in a location not meant to be covered by a socket, or if they are close in close proximity to one another, which might cause interference during signal pick-up using surface EMG electrodes.

Here, we present for the first time the concept of high-level upper limb amputation and subsequent bionic substitution of the entire upper extremity in patients with global brachial plexus avulsion injuries and lack of treatment alternatives. The surgical procedures that were performed to improve the biotechnological interface and facilitate prospective prosthetic control are described in this article. After transhumeral or glenohumeral amputation, patients controlled the prosthetic arm with EMG signals picked up from muscles in the upper arm and/or muscles around the shoulder girdle. Final outcome measures are presented including effects on upper limb function, deafferentation pain, subjectively perceived disability and quality of life. Pitfalls of and contraindications for bionic reconstruction are discussed for this highly specific patient population. 

## 2. Materials and Methods

For the conduction of this study, ethics approval was obtained from the ethics committee of the Medical University of Vienna, Austria (Ethical vote number: 1009/2014). All patients gave written informed consent.

### 2.1. Patient and Demographic Data

**Figure 1:**
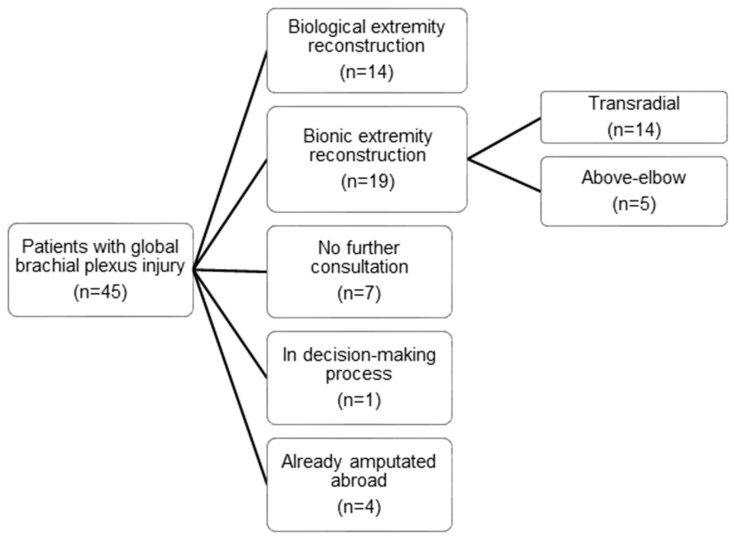
Flowchart showing detailed reasons for exclusion of patients with a global brachial plexus injury from the study.

Between 2012 and 2017, forty-five patients with global brachial plexus injuries approached our centre for a primary consultation (Figure 1). A global brachial plexus injury was defined as an avulsion of at least three of five nerve roots and/or substantial damage to the supra- and infraclavicular parts of the brachial plexus (BP), therefore affecting the sensorimotor function of the entire upper extremity. Of these, 31.1% (*n* = 14) received reconstructive surgery with the goal to improve biological upper limb function, while 15.6% (*n* = 7) did not return after initial consultation and 8.9% (*n* = 4) had already been amputated upon their first visit. Nineteen patients (42.2%) were treated with bionic reconstruction, of which fourteen (31.1%) were amputated at the transradial level and were therefore excluded from this study.

Therefore, five patients with complete brachial plexus injury (BPI) who underwent bionic reconstruction after elective high-level upper limb amputation were enrolled in this study.

All five patients were male with a mean age of 37.5 ± 12.3 years (range 24.0–45.1 years) at primary consultation. In four of five patients, motorcycle accidents were the cause for the extensive BPI. The multiple nerve root avulsions ranged from three (*n* = 1) and four roots (*n* = 2) to all five roots (*n* = 2), always including C7 to T1. In all five patients, primary brachial plexus (BP) reconstruction was performed elsewhere before initial consultation at our institution (Table 1), with a time delay of 4.4 ± 0.9 months (range 3–5 months) between BPI and primary reconstruction; however, no functional improvement was achieved for the hand and elbow. The average time interval between trauma and initial consultation at our institution was 9.0 ± 7.5 years (range 1.0–20.8 years). 

### 2.2. Surgeries Performed to Improve the Biotechnological Interface

**Figure 2:**
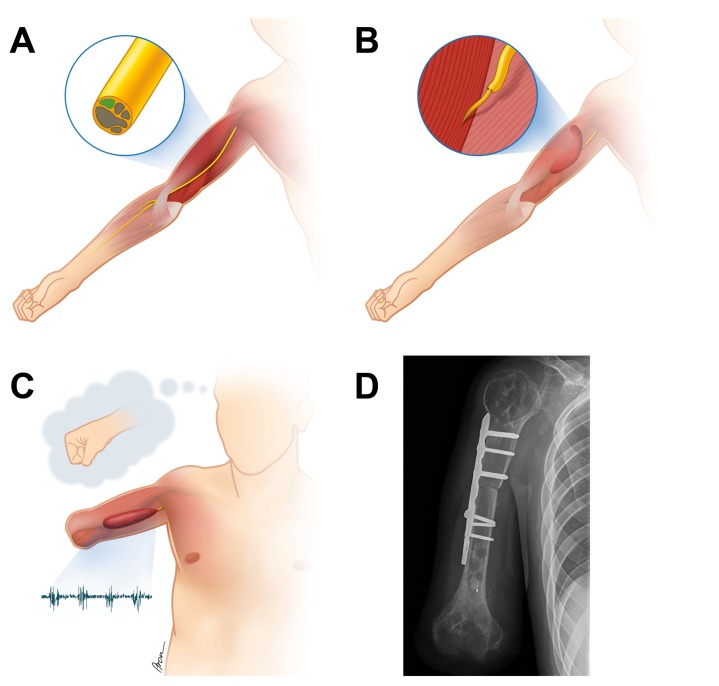
(**A**) Despite complete muscle atrophy in the patient’s forearm a fascicle group containing viable motor axons was identified in the median nerve with an intra-operative fast staining method screening for acetylcholine positivity. (**B**) A free functional muscle, i.e., the gracilis muscle from the patient′s leg, was transferred to the medial upper arm and its muscle nerve branch (the obturator nerve) was co-apted to the fascicle group previously tested positive for the presence of functional motor axons. (**C**) After successful nerve regeneration and elective amputation, the patient′s attempt to make a fist produced a reliable EMG signal detectable with transcutaneous electrodes placed over the muscle. (**D**) To improve future prosthetic handling and avoid excess length of the prosthetic limb, a humerus shortening osteotomy was performed upon amputation in the same patient.

**Figure 3:**
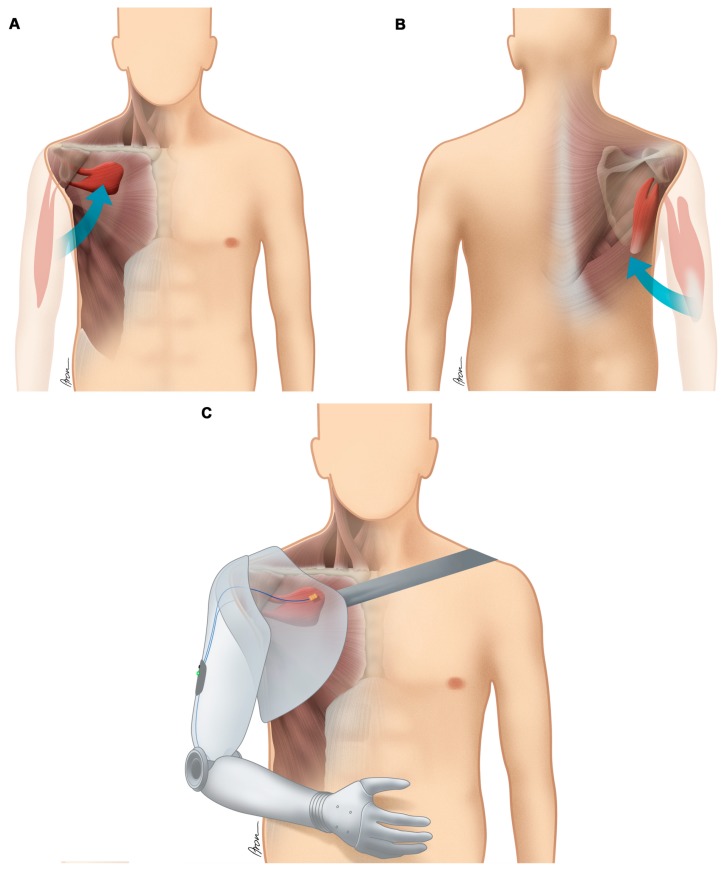
Adaptation of the human anatomy to improve the biotechnological interface and the information transfer between man and machine as performed in Case No. 3. To preserve valuable EMG activity, (**A**) the biceps muscle was transferred to the infraclavicular fossa and (**B**), the triceps muscle was transferred to the infraspinatous fossa. (**C**) The patient now controls his prosthetic arm with a two-signal control (transferred biceps and triceps m.); co-contraction of both signals allows him to switch between the three degrees of freedoms (elbow flexion/extension, hand rotational unit, hand opening/closing).

Table 2 lists the muscle activity for each patient detected with sEMG sensors in the upper arm and/or shoulder girdle upon initial consultation; the surgeries, which have been performed to improve the communication of the patient′s body with the mechatronic device; and the level of amputation. Figure 2 illustrates the surgical procedures performed in Case No. 1, including a free functional muscle transfer and humerus shortening osteotomy. The rearrangement of muscles in the context of biotechnological interfacing at the time of elective amputation is schematically exemplified for Case No. 3 in Figure 3.

### 2.3. Outcome Measures

#### 2.3.1. Functional Outcome Measures

Global upper extremity function using two objective functional tests was assessed at three time points: before bionic reconstruction using the functionless arm; shortly before amputation using a hybrid prosthetic arm [15,16] mounted on the functionless arm of the patient; and after successful prosthetic reconstruction.

Functional assessment instruments included the Southampton hand assessment procedure (SHAP), and a modified action research arm test (ARAT). 

The SHAP consists of tasks involving heavy and light object manipulation and fourteen activities of daily living (ADL), with participants self-timing their speed to complete each task [20]. Normal hand function is indicated by a score of ≥100 points. The ARAT consists of four sections with different manual tasks and has a score maximum of 57 points [21]. While it is usually performed with the patient sitting in front of the test kit on a table, here, patients were allowed to stand in order to avoid a flooring effect due to limited shoulder range of motion in this specific patient population.

Subjective disability resulting from the upper extremity injury as perceived by the patient was evaluated with the disabilities of the arm, shoulder and hand (DASH) questionnaire. The DASH is a patient-centred questionnaire and consists of thirty items evaluating subjectively perceived disability in activities of daily living when using both arms/hands [22]. A score of 100 indicates the worst and 0 indicates the best hand function.

#### 2.3.2. Pain Assessment

Pain scores relevant to the affected plexus or phantom hand were evaluated using a 10-point visual analogue scale (VAS) [23]. 

#### 2.3.3. Patient-Reported Quality of Life Assessment

The SF-36 Health Survey was used to evaluate the patients′ quality of life before and after bionic reconstruction. The 4-week recall version of the questionnaire was used. The questionnaire consists of 36 items, which measure the following eight subscales: limitations in physical activities because of physical health problems (subscale physical functioning), limitations in usual role activities because of physical health problems (subscale role—physical), bodily pain (subscale bodily pain), general health perceptions (subscale general health), energy and fatigue (subscale vitality), limitations in social activities because of physical or emotional problems (subscale social functioning), limitations in usual role activities because of emotional problems (subscale role—emotional), and psychological distress and well-being (subscale mental health) [24]. 

Based on these eight subscales, two superior physical and mental component summary scales are calculated [21]. All subscales and summary scales have mean values of 50 with a standard deviation of 10, with calculated *T*-value scores above 60 indicating above average health and scores below 40 indicating below average health compared against published age- and sex-matched norm samples.

#### 2.3.4. Statistical Analysis

The results of the outcome measures are presented as absolute values, mean and standard deviation. The differences between the variables before and after bionic reconstruction were reviewed graphically (Q-Q plot) and tested with the Shapiro-Wilk test. Since all the differences could be considered normally distributed, they were analysed with paired, one-sided Student′s *t*-tests. 

The significance level alpha for all implemented tests was set to α < 0.05. To counteract the problem of multiple comparisons Bonferroni–Holm correction was used and the corrected *p*-values are presented. The statistical analysis was performed using IBM SPSS version 24.

## 3. Results

Mean follow-up time for the five patients enrolled in this study was 19.2 ± 8.2 months (range, 11–33 months). While functional assessments with a hybrid prosthetic arm were completed for all patients, final functional outcome measures were obtained in three of five patients only. Although Case No. 1 showed reliable and distinct myosignals he refused to wear a prosthesis due to hyperhidrosis, the weight of the device and discomfort in the socket. The patient rejected therapy to improve prosthetic control and did not agree to have his socket adjusted. After selective nerve transfer surgery and elective amputation, Case No. 2 was fitted with a prosthetic arm abroad and did not return to our centre for a final functional follow-up assessment. 

Table 3 lists individual results for all outcome measures obtained in the five study patients except for SF-36 Health Survey data (see below). Video S1 (Supplemental Material) shows all three functional assessments in Case No. 5 (with the plexus arm, the hybrid arm and the final prosthetic arm) and can be found online [25].

### 3.1. Functional Outcome 

**Figure 4:**
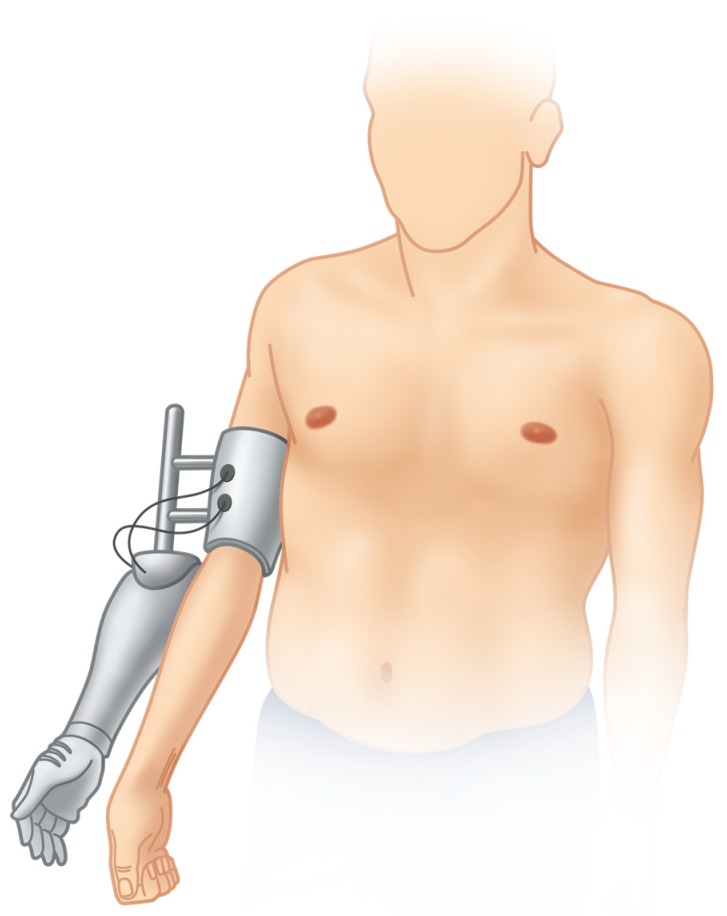
After initial rehabilitation including surface EMG signal training a hybrid prosthetic arm is mounted onto the functionless plexus arm. The patient controls it with the EMG signals identified and trained previously. This allows a prediction of future prosthetic control. The results of the objective hand function tests using the hybrid arm are video-documented and need to be superior to original upper extremity function before elective amputation of the plexus arm may be considered.

Using a hybrid prosthetic arm (Figure 4), mean ARAT scores significantly improved from 0.6 ± 1.3 to 11.0 ± 6.7 (*p* = 0.042), while mean SHAP scores also improved from 4.0 ± 3.7 to 13.8 ± 9.2, however not significantly (*p* = 0.058). Mean ARAT and SHAP scores in three patients using the final prosthetic arm further improved to 17.3 ± 1.5 and 22.0 ± 9.2, respectively. Due to the small sample size (*n* = 3) statistical testing was not applied here.

Subjectively perceived disability as measured with the DASH questionnaire significantly decreased from 52.5 ± 9.4 before treatment to 31.2 ± 9.8 (*p* = 0.003) after final prosthetic fitting. 

### 3.2. Pain

Mean VAS scores evaluating deafferentation pain in the affected upper limb decreased from 8.5 ± 1.0 to 6.7 ± 2.1 (*p* = 0.055), however not significantly.

### 3.3. Patient-Reported Quality of Life

Individual results for all five patients are listed in Table 4. The mean physical component summary scale after bionic reconstruction improved from 32.9 ± 6.4 to 40.4 ± 9.4 (*p* = 0.058), while the mental component summary scale increased significantly from 43.6 ± 8.9 to 57.3 ± 5.5 (*p* = 0.021).

## 4. Discussion

Global brachial plexus avulsion injuries have a profound impact on daily activities as a result of the loss of upper limb function [26] and may also cause severe psychological distress due to socio-economic hardship [27]. Here, we have shown that prosthetic rehabilitation after bionic substitution of the entire arm enables useful upper limb function in patients with global brachial plexus avulsion injuries, where biological treatment alternatives have failed to improve function. 

Bionic reconstruction may involve surgeries to improve the biotechnological interface between man and machine. One goal is to increase the number of available EMG signals for prosthetic control. Many patients with global brachial plexus avulsion injuries are not capable of voluntarily activating single muscles in their affected upper limb. During structured rehabilitation the patients need to re-learn how to specifically address single muscles. This is highly cognitively demanding since faint muscle contractions do not lead to actual movements of the arm and hand and the brain has long “lost” the central representation of the denervated extremity. In some patients intense cognitive training including motor re-learning with appropriate biofeedback [28,29,30] result in the identification of separable sEMG signals. In others, new muscles need to be added (free functional muscle transfer) or relocated to establish sEMG signals available for prosthetic control. In all, at least two signals are sufficient for solid prosthetic use. The addition or surgical creation of a third signal with the goal of improved prosthetic control needs to be considered for each patient individually, depending on biological prerequisites and patient expectations. 

When surgically establishing control signals in patients with global brachial plexus avulsion injuries, the focus should be to create a limited number of stable myoelectric sites, to achieve cognitively simple and robust control. As patients after BPI will have difficulties to alternately contract different muscles due to aberrant re-innervation and functional “confusion” in the reconstructed nerves, a challenging prosthetic control algorithm based on a complex array of myosignals most probably will lead to frustration, malfunction and abandonment of the prosthetic device. Therefore, it is our conviction, that only cognitively “simple” control strategies should be applied in this particular patient population. Furthermore, given the sparse and “confused” neurological input to the muscles in BPI patients, even simple two-signal prosthetic control requires a decent amount of therapy including sEMG biofeedback training to establish reliable control [28,30]. Case No. 4, for example, did not receive surgery to create an additional EMG signal because he already had two reliable signals at shoulder level. With these, simple prosthetic movements were possible after intense cognitive signal training as demonstrated with the hybrid prosthetic arm. He was satisfied with this functional improvement and we thus decided not to perform additional muscle and nerve transfers for this specific case. 

If insufficient numbers of EMG signals are available due to extensive muscle degeneration, free functional muscle transfers can be performed to establish additional EMG signal sites given that viable motor axons are identified for nerve coaptation. In our laboratory an intra-operative fast staining technique was developed (unpublished data, Gesslbauer et al.), which screens nerve biopsies for acetylcholine positivity indicating the presence of live motor axons. If fascicles containing functional motor axons are identified these may be used to re-innervate a transferred, “healthy” muscle (see Figure 2A,B). The gracilis and adductor longus muscles have been used in the context of bionic reconstruction. For reliable signal control, voluntary muscle activation should produce a sEMG signal that repeatedly has an amplitude 2–3 times higher than its amplitude during relaxation [30]. After nerve regeneration, the signals’ strength and their activation patterns are regularly observed during training using sEMG biofeedback to allow optimal functional outcomes at the end of the prosthetic fitting.

Transhumeral and glenohumeral amputees are more likely to report discontinuation of prosthetic use compared to more distal amputations [31]. Critical factors include unphysiologic weight distribution of the prosthetic arm, socket design with cumbersome constructions mounting the artificial limb on the patient body, and lack of sensory feedback [32]. Hence, amputees often do not experience sufficient improvement in their daily life using the prosthetic device, which consequently results in high rates of abandonment of up to 50% [31,33,34]. However, prosthetic limb replacement in high-level amputees is of particular importance when considering problems with posture and balance, which are often present in these patients [35,36]. 

Our hypothesis was that device abandonment may be reduced by facilitating the information transfer between patient body and prosthetic device through surgical procedures aimed at improving the biotechnological interface. As shown in this report, different strategies may be employed (Table 2, Figure 2 and Figure 3). First and foremost, patient expectations must be considered when initiating bionic reconstruction. The level of amputation (transhumeral, glenohumeral) needs to be thoroughly discussed with the patient depending on neurobiological prerequisites (detected myoactivity) as well as the patient′s preferences. In long-standing, global brachial plexus avulsion injuries a subluxation of the glenohumeral joint due to denervation of the rotator cuff is often present [37]. Disabling shoulder pain, which has to be differentiated from deafferentation pain in the rest of the arm and hand, before and after amputation needs to be taken into account when planning the level of amputation and designing the final prosthetic socket, which should ideally counteract problems like an unstable shoulder joint. A disarticulation of the joint or short transhumeral stump creation (as explicitly requested by some patients) can be met with surgical re-arrangement of the muscle signals in the shoulder girdle and prosthetic socket design using a harness including the contra-lateral shoulder, which will distribute the weight from the prosthetic arm to the upper trunk, thereby improving day-to-day wearing comfort (see Figure 3). Additionally, realistic outcomes need to be discussed and a standardized psychological screening should evaluate capability and mental strength to undergo the procedure as well as the absence of psychiatric contra-indications, as previously defined [38].

As stated before, simple control mechanisms are the key element of successful prosthetic rehabilitation in brachial plexus patients as they embrace their prosthetic arm as an assistive device during daily life activities. While “transradial” bionic patients make use of their prosthetic hand for the most part of the day, patients undergoing bionic substitution at a more proximal level will use it for specific activities only, liberating the healthy hand for small-object manipulation. Such simple tasks include carrying a shopping bag, holding or stabilizing an object, and opening a door. The simple control of the prosthetic device should be given priority to prevent its abandonment due to lack of reliability. 

Recently, it has been shown that bionic reconstruction has the potential to reduce deafferentation pain in BP avulsion patients [15,38]. With growing numbers of patients, we have recognized that pain reduction has great inter-individual variations. Although most patients showed reduced VAS scores in the present study, this difference was not statistically significant (*p* = 0.055). Case No. 1, who experienced the most disabling pain at initial consultation (VAS 10.0), did not report of any pain reduction at final follow-up (VAS 9.8). This patient refused to wear his prosthetic arm after final fitting due to hyperhidrosis, excessive weight of the prosthesis and discomfort in the socket. While therapy to improve control and socket adjustment were both offered to the patient, he denied further treatment. Interestingly, he stated that the amputation itself had had a positive impact on his life as was also reflected by his test results (improved DASH score and SF-36 subscale scores, i.e., physical, emotional and social functioning). On the other hand, Case No. 4 reported that phantom limb pain in the arm and hand may decrease to a minimum when he is fully engaged in bimanual tasks and wears his prosthesis on average ten hours per day. After doffing the device, pain levels dramatically increase again at night-time without proper pain medication. To date we may conclude that prosthetic limb replacement in brachial plexus avulsion patients has the potential to reduce deafferentation pain, with fluctuating inter-individual results and some patients benefitting more than others. A long-term study evaluating stump pain, phantom limb pain, and individual prosthesis wearing rates is needed to further elucidate its effect on deafferentation pain.

While in all five patients most SF-36 subscale scores were below average at initial consultation (cut-off <40, see Table 4), limitations in physical activities because of physical health problems, vitality as well as social and emotional functioning were improved after bionic reconstruction in the majority of patients to normal or above-average levels. The mean mental component summary scale significantly improved from 43.6 ± 8.9 to 57.3 ± 5.5 (*p* = 0.021). Patients generally reported that participation in social activities was dramatically improved after bionic arm substitution. 

The choice of amputation level in BP patients who qualify for prosthetic reconstruction depends primarily on the remaining ability of elbow flexion and the presence of myosignals in the forearm. However, the particular preferences of the patient need to be considered as well. Even after surgical optimization of the biotechnological interface, above-elbow amputation will generally lead to more cumbersome prosthesis handling and inferior functionality compared to the transradial level. Some patients may choose not to wear the device because of these limitations (see Case No. 1). Nonetheless, our experience shows that after careful patient selection the loss of the non-functional limb alone is perceived as a relief for most. Systematic and thorough psychosocial assessment is key in order to avoid rash decisions at this point of no return (39). 

Internal validity of the study is limited due to the small sample size studied. Brachial plexus avulsion injury is a very rare condition and patients who do not regain any function in their affected upper limb despite primary and secondary reconstructive attempts and who are suitable for bionic reconstruction are even less common. Being the only centre worldwide that offers this procedure therefore makes the recruitment of higher patient numbers visionary at the current moment. 

## 5. Conclusions

Here, we report for the first time that high-level elective amputation and bionic reconstruction may be applied in global brachial plexus avulsion injuries to restore arm and hand function. Simple but robust upper limb function may be restored without timely limitation after the accident when biological treatment alternatives have failed. Quality of life and subjectively perceived disability is thereby improved in these patients.

## Figures and Tables

**Table 1:** Type of accident, lesion and timely primary reconstructive surgeries performed elsewhere before initial consultation at our institution.

Case No.	Type of Accident	Type of Lesion, Side	Primary Reconstructive Surgeries aimed at Restoration of Shoulder and Elbow Function
**1**	Motorcycle	Avulsion of roots C5-T1, right	Transfer of accessory nerve to suprascapular nerve and hypoglossal nerve to MCN resulted in a stable shoulder; elbow function did not recover (surgery performed elsewhere five months after injury)
**2**	Work-related injury	Rupture of roots C5-C6 and avulsion of C7-T1, left	Sural nerve grafts were used to bridge the defects of C5 and C6 to restore elbow flexion and shoulder stability; motor recovery was unable to move the biological arm (surgery performed elsewhere five months after injury)
**3**	Motorcycle	Avulsion of roots C6-T1, right	Restoration of elbow function was attempted with ICN transfers to MCN, however no recovery was achieved (surgery performed elsewhere five months after injury)
**4**	Motorcycle	Avulsion of roots C5-T1, left	Transfer of phrenic to suprascapular nerve and ICN transfers to MCN and axillary nerve resulted in a stable shoulder; elbow function did not recover (surgery performed elsewhere four months after injury)
**5**	Motorcycle	Avulsion of roots C6-T1, right	Restoration of elbow function was attempted with nerve grafts from C4 and C5 to MCN; ICN transfers to median nerve; motor recovery was unable to move the biological arm (surgery performed elsewhere three months after injury)

ICN = intercostal nerve, MCN = musculocutaneous nerve.

**Table 2:** Surface electromyographic signals at initial consultation for all five patients and surgeries performed to improve the man-machine interface.

Case No.	sEMG Signal Sites at Initial Consultation	Surgeries Performed to Improve the Biotechnological Interface	Level of Amputation
**1**	biceps m. + triceps m.	free gracilis muscle transferred to medial upper arm and neurotization of median nerve to obturator nerve to generate a third EMG signal; humerus shortening osteotomy	elbow ex-articulation (with humeral shortening osteotomy)
**2**	no detectable myoactivity in the upper arm	free gracilis muscle transferred to dorsal upper arm and neurotization of thoracodorsal nerve to obturator nerve; free adductor longus muscle transferred to medial upper arm and neurotization of median nerve to obturator nerve	transhumeral
**3**	biceps m. + triceps m.	transfer of pedicled biceps and triceps muscles to infaclavicular fossa and infraspinatus fossa	glenohumeral
**4**	infraspinatus m. + pectoralis major m.	ND	glenohumeral
**5**	pectoralis major m. + biceps m. + brachioradialis m.	transfer of pedicled brachioradialis muscle to dorsal upper arm to preserve this signal site upon elective transhumeral amputation	transhumeral

m = muscle; ND = not done; sEMG = surface electromyographic.

**Table 3:** Outcome measures including functional testing with the plexus arm, hybrid arm and prosthetic arm, as well as DASH scores and pain scores before and after bionic reconstruction.

	ARAT			SHAP			DASH		VAS	
Case No.	Before	Hybrid	After	Before	Hybrid	After	Before	After	Before	After
1	0	17	ND	7	10	ND	57.5	36.7	10	9.8
2	3	16	ND	6	18	ND	37.5	15	8.2	6.9
3	0	0	17	0	24	30	49.2	34.2	7.8	6.5
4	0	11	19	0	0	12	60	30	7.5	4
5	0	11	16	7	17	24	58.3	40	9.1	6.4
MEAN ± SD	0.6 ± 1.3	11.0 ± 6.7	17.3 ± 1.5	4.0 ± 3.7	13.8 ± 9.2	22.0 ± 9.2	52.5 ± 9.4	31.2 ± 9.8	8.5 ± 1.0	6.7 ± 2.1

In ARAT and SHAP, higher scores refer to better upper extremity function. The maximum score for ARAT is 57 and in SHAP normal hand function is regarded as equal to or above 100 points. In DASH lower scores are desirable, with 100 indicating the worst and 0 indicating the best hand and arm function. ARAT = action research arm test, DASH = disabilities of the arm, shoulder and hand, ND = not done, VAS = visual analogue scale, SHAP = Southampton hand assessment procedure.

**Table 4:** Individual test results of SF-36 health survey for all five patients including the eight independent subscales and two superior component summary scales.

	Case No.	Physical Functioning	Role Physical	Bodily Pain	General Health	Vitality	Social Functioning	Role Emotional	Mental Health	Physical Comp. Sum. Scale	Mental Comp. Sum. Scale
**Before amputation**	**1**	28	10	18	56	38	32	36	48	37.8	48.2
	**2**	42	20	26	64	44	41	12	50	38.6	44.3
	**3**	39	29	34	54	44	48	39	52	36.3	54.5
	**4**	28	22	34	17	26	10	28	20	26.3	30.8
	**5**	0	0	14	38	24	24	18	35	25.6	40.1
	**MEAN ± SD**	27.4 ± 16.6	16.2 ± 11.3	25.2 ± 9.1	45.8 ± 18.7	35.2 ± 9.7	31.0 ± 14.8	26.6 ± 11.5	41.0 ± 13.5	32.9 ± 6.4	43.6 ± 8.9
**After bionic reconstruction**	**1**	44	52	33	56	40	54	53	52	49.7	54.2
	**2**	47	54	40	50	58	55	53	55	46.1	59.4
	**3**	44	38	37	62	52	55	53	60	40.7	62.9
	**4**	40	22	37	20	32	26	54	42	25	49.4
	**5**	14	52	33	44	48	46	53	60	40.5	60.8
	**MEAN ± SD**	37.8 ± 13.5	43.6 ± 13.7	36.0 ± 3.0	46.4 ± 16.2	46.0 ± 10.2	47.2 ± 12.4	53.2 ± 0.4	58.8 ± 7.4	40.4 ± 9.4	57.3 ± 5.5

comp. sum. = component summary scale.

## Supplementary Materials

The following is available online at https://www.mdpi.com/2077-0383/9/1/23/s1. Video S1: Functional assessment of Case No. 5 with the plexus arm, the hybrid arm and the final prosthetic arm.
